# Regional gray matter correlates of memory for emotion-laden words in middle-aged and older adults: A voxel-based morphometry study

**DOI:** 10.1371/journal.pone.0182541

**Published:** 2017-08-03

**Authors:** Carina Saarela, Juho Joutsa, Matti Laine, Riitta Parkkola, Juha O. Rinne, Mira Karrasch

**Affiliations:** 1 Department of Psychology, Abo Akademi University, Åbo, Finland; 2 Centre for Cognitive Neuroscience, University of Turku, Turku, Finland; 3 Turku PET Centre, Turku University Hospital, Turku, Finland; 4 Department of Neurology, University of Turku, Turku, Finland; 5 Athinoula A. Martinos Center for Biomedical Imaging, Massachusetts General Hospital and Harvard Medical School, Boston, MA, United States of America; 6 Berenson-Allen Center for Noninvasive Brain Stimulation, Beth Israel Deaconess Medical Center and Harvard Medical School, Boston, MA, United States of America; 7 Turku Brain and Mind Center, University of Turku, Turku, Finland; 8 Department of Radiology, University of Tampere, Tampere, Finland; 9 Department of Radiology, Tampere University Hospital, Tampere, Finland; 10 Division of Clinical Neurosciences, Turku University Hospital, Turku, Finland; University of Akron, UNITED STATES

## Abstract

Emotional content is known to enhance memory in a content-dependent manner in healthy populations. In middle-aged and older adults, a reduced preference for negative material, or even an enhanced preference for positive material has been observed. This preference seems to be modulated by the emotional arousal that the material evokes. The neuroanatomical basis for emotional memory processes is, however, not well understood in middle-aged and older healthy people. Previous research on local gray matter correlates of emotional memory in older populations has mainly been conducted with patients suffering from various neurodegenerative diseases. To our knowledge, this is the first study to examine regional gray matter correlates of immediate free recall and recognition memory of intentionally encoded positive, negative, and emotionally neutral words using voxel-based morphometry (VBM) in a sample of 50-to-79-year-old cognitively intact normal adults. The behavioral analyses yielded a positivity bias in recognition memory, but not in immediate free recall. No associations with memory performance emerged from the region-of-interest (ROI) analyses using amygdalar and hippocampal volumes. Controlling for total intracranial volume, age, and gender, the whole-brain VBM analyses showed statistically significant associations between immediate free recall of negative words and volumes in various frontal regions, between immediate free recall of positive words and cerebellar volume, and between recognition memory of positive words and primary visual cortex volume. The findings indicate that the neural areas subserving memory for emotion-laden information encompass posterior brain areas, including the cerebellum, and that memory for emotion-laden information may be driven by cognitive control functions.

## Introduction

The emotional enhancement effect of memory (EEM) entails the augmentation of the formation and strength of memory traces for emotion-laden information [1–3]. The EEM is preserved over the life span in healthy adults [4], even though the general level of working memory and episodic memory functioning may decline [5]. However, with increasing age, qualitative changes in the preferences for emotion-laden information have been observed [6–8]. The preferences relate to the two basic bidirectional dimensions of emotion according to the circumplex theory of emotion [9]: emotional valence, or whether a stimulus is perceived as positive or negative, and emotional arousal, or whether a stimulus is perceived as calming or exciting. The age-related shift in preferences has been coined the positivity effect [10]. In younger adults, a negativity bias, indicating a relative preference for negative over positive information, has been commonly observed [8, 11–13]. In middle-aged and older adults, the negativity bias has been found to be reduced [13–14], if not replaced by a positivity bias, i.e., a relative preference for positive over negative information [8, 12]. The positivity effect seems to be modulated by arousal, as the age-related valence-specific differences in memory were observed for low-arousing stimuli, but not for high-arousing stimuli [12]. However, the positivity effect has not been consistently demonstrated. In a recent meta-analysis of 100 studies, Reed et al. (2014) showed that it was most likely to be found in studies with wider age comparisons and in studies that did not impose experimental constraints on cognitive processing, such as using intentional encoding instructions as opposed to an incidental encoding paradigm [8].

Much effort has been devoted to behavioral studies on the EEM and the positivity effect, but studies on the neuroanatomical correlates of memory for emotion-laden stimuli in middle-aged and older healthy adults are very few. In the present study, we sought to examine associations between immediate free recall and recognition memory of emotion-laden words and regional gray matter volume in a sample of 50-79-year-old cognitively intact adults. Therefore, the literature review on age-related neural correlates of memory for emotion-laden stimuli will focus on this particular age range.

Lesion studies [15–16] and functional neuroimaging studies [17–22] on the neural underpinnings of emotional memory processes have so far identified an extensive network of cortical and subcortical brain areas that are involved in general as well as specific task performance. The main function of this neural network appears to be to link emotions to stimulus events [23]. The network is commonly thought to comprise the amygdalae, the hippocampi, the medial and lateral prefrontal cortices (PFC), and the basal ganglia [23]. These brain areas are extensively interconnected [24–25], and also linked to the sensory cortices [25–26]. The functional implications of the connections and projections are not yet fully understood, but it is clear that the brain areas involved in processing emotion-laden information are also involved in processing non-emotion-laden information [27–28].

The memory modulation hypothesis by McGaugh (2000) states that amygdalar activation during memory processing of emotion-laden information exerts a modulatory effect by boosting the processing of information with survival value [29]. The amygdalae modulate the activity in other brain areas subserving cognitive processing especially via interaction with the adrenergic system [1–2, 21, 29–30]. This takes place through the extensive anatomical connections that the amygdalae have with many parts of the brain, such as the orbitofrontal cortex (OFC), the anterior cingulate cortex (ACC), the ventral striatum, the hippocampi, and the occipital cortex [1–2, 21, 24–26]. Amygdalar modulation of the activity of the hippocampal formation during memory formation of emotional events is seen as a necessary prerequisite for memory consolidation [2, 29], as patients with amygdalar damage have failed to produce EEM [15]. Amygdalar activation has been observed during encoding [13, 17, 19–22, 30] as well as retrieval of emotion-laden stimuli [18, 31], indicating that the amygdalae mediate the EEM not only during encoding through increased attention and elaboration, but also during consolidation and retrieval, by enhancing the consolidation of memory traces [2]. Alternatively, the retrieval-related activation may be interpreted as a part of the contextual information associated with an event, which then functions as a cue to enable the successful retrieval of that event [23]. However, the amygdalae are not considered the loci of the EEM, as the enhancement effect in itself is thought to occur within or be mediated by the hippocampi [29].

Several prefrontal areas have been associated with functionally specific contributions to the EEM [13, 17–21]. These areas mainly include the orbitofrontal cortex (OFC) and the ventromedial PFC [28], but also the dorsolateral PFC [32–33]. The ventromedial PFC involves the medial OFC and the ventral and rostral ACC [34]. The prefrontal brain areas have reciprocal connections with virtually every sensory system, with cortical and subcortical motor systems, and with limbic and midbrain structures involved in memory and emotion [35]. Furthermore, the various areas of the PFC are extensively interconnected [35].

Whereas the role of the amygdalae in the EEM is to help boost memory for emotion-laden stimuli through bottom-up automatic processing of their survival value in terms of the emotional arousal that they evoke, the PFC seems to contribute via top-down controlled processing of their value in terms of emotional valence [21, 36], specifically in relation to the self [37]. The OFC is assumed to take part in the EEM by integrating exteroceptive and interoceptive sensory information to guide behavior [27]. The OFC and the medial PFC engage together in the processing of value in stimuli [33, 35, 38] and the computation of outcome expectancies [28]. The ventromedial and dorsomedial PFC participate in the detection of self-relevant stimuli [38–39] and in self-reflection [34, 38]. The lateral PFC, particularly the dorsolateral part, contributes to the cognitive control of emotion [32–33, 40–41] through its engagement in top-down, goal-directed selection of responses [32–33, 35, 41], the explicit evaluation of stimuli [27], working memory [42], and control of attention [43], mainly accomplished by its reciprocal connections with the OFC and the medial PFC [24]. Because of its involvement in these higher-order cognitive functions, the role of the dorsolateral PFC in emotional processing is suggested to be of a general nature [28].

There is an abundance of functional neuroimaging studies on the neural underpinnings of the emotional memory processing in younger adults, but only a few studies have been published regarding age-related functional or structural brain differences in younger versus older healthy adults concerning the EEM and valence-specific memory performance. The functional neuroimaging studies have revealed age-related differences in the strength of activation in areas consistently implicated in the EEM in younger adults, and also in the activation loci [13, 19], even in a valence-specific manner [13]. This suggests some degree of age-related specificity in the neural substrates of memory for emotion-laden stimuli. Whereas functional neuroimaging studies typically focus on the most reliable activation loci across individuals, thus removing variability in behavior and brain functionality by averaging, structural neuroimaging studies reveal how variability in structure is related to inter-individual differences in behavior [44]. Studying the regional gray matter volumetric correlates of memory performance in middle-aged and older adults should therefore be especially fruitful, as increasing age seems to bring increased variability in both measures [45–47]. Naturally, limiting the age range to the later years of adulthood precludes the study of age-related specificity of the structural brain correlates of memory for emotion-laden stimuli. It is known that gray matter volumetric correlates of behavior reflect the age of the participants [48]. This has been taken to indicate that the microstructural mechanisms underlying regional gray matter volume as measured by voxel-based morphometry (VBM) may be age-specific [44, 48]. Therefore, it is plausible to assume that the gray matter volumetric correlates of memory for emotion-laden words may be different in young adults.

Most of the structural neuroimaging studies on regional gray matter correlates of memory for emotion-laden stimuli or events have focused on the amygdalae and the hippocampi [49–53]. Whole-brain volumetric correlates have been examined to a lesser extent, and so far only in combined groups of normally aged adults and patients suffering from amnestic mild cognitive impairment [54], Alzheimer’s disease [54–56], and variants of frontotemporal dementia [55–56]. Findings pertaining to associations between amygdalar volume and memory for emotion-laden stimuli in middle age and older age have been mixed. Some studies have demonstrated no associations in middle-aged and older healthy adults [49–50, 51] or in patients suffering from Alzheimer’s disease [49], while others have demonstrated the expected positive correlations in combined groups of patients and healthy controls [54–56] or in patients with neurodegenerative diseases [50–52]. Interestingly, associations between memory for neutral stimuli and amygdalar volumes have also been reported [53–54], although not consistently [50].

A slightly different pattern can be discerned for hippocampal volumetric associations with memory for emotion-laden stimuli in middle age and older age. Positive correlations with memory for emotion-laden stimuli have been observed in normal aging [49], in neurodegenerative diseases [49–50, 52–53], and in combined groups of patients and healthy controls [49, 54, 56], albeit not consistently [50, 55]. Similarly, mixed findings can be seen for the associations with memory for emotionally neutral stimuli [50, 53–54].

The studies examining whole-brain gray matter correlates of memory for emotion-laden stimuli in combined groups of patients with neurodegenerative disorders and normally aged controls have revealed that larger gray matter volume in the OFC and ventromedial and ventrolateral PFC was correlated with better memory for negative stimuli [54–55]. Mistridis et al. (2014) also included positive stimuli in their study, and found immediate free recall of positive words to be related to gray matter volume in one cluster centered in the left angular gyrus, extending into the middle temporal gyrus [54]. Delayed free recall of positive words was associated with gray matter volume in a cluster centered in the left hippocampus, extending into the amygdala, the perirhinal, entorhinal and parahippocampal cortices, and the lingual gyrus [54].

All in all, previous studies on regional gray matter correlates of memory for emotion-laden stimuli in middle and older adulthood have either focused on amygdalar and hippocampal volumetric associations [49–53], or—when examining whole-brain associations—studied these correlates in heterogeneous groups including both patients with neurodegenerative disorders and normally aged controls [54–55]. The studies on amygdalar volumetric associations have found no correlations in middle-aged and older healthy adults, which may be due to a lack of sufficient statistical power to detect subtle associations as the samples have included 20 participants at the most [50, 53]. Age-related decline in amygdalar volume is considered to be relatively less notable than in other brain regions, such as the hippocampi [57], although the findings are mixed. For example, Fjell et al. (2009) demonstrated a similar rate of age-related decline in both structures [58]. In the present study, the sample consisted of 46 individuals and the age range was wider than in previous studies, enabling more variance in both volumetric and memory measures. Also, this study is to our knowledge the first one to examine these associations in middle-aged and older healthy adults using word stimuli, and to examine whole-brain regional gray matter correlates of memory for positive and negative stimuli, respectively, in healthy adults only. A different pattern of results compared to the previous studies on whole-brain gray matter correlates is to be expected, as Kumfor et al. (2013) demonstrated condition-related differences in the neural contributions to recognition memory of negative stimuli in groups of patients suffering from Alzheimer’s disease or variants of frontotemporal dementia [55]. Together with the fact that the behavioral results for emotional memory also differed between the patient groups, it seems likely that different neurobiological mechanisms were responsible for the divergent behavioral profiles in the patient groups [55]. It may thus be that different structural brain correlates may underlie memory for emotion-laden stimuli in middle-aged and older healthy individuals without neurodegenerative disorders. This possibility is also suggested by functional neuroimaging studies revealing age-related specificity in the neural underpinnings of memory for emotion-laden stimuli [13, 19]. Furthermore, as functional neuroimaging studies [13] and regional gray matter volumetric studies [50, 54] using both negative and positive stimuli have demonstrated valence-specific neural correlates of memory for emotion-laden stimuli in older adults, we wanted to study the neuroanatomical contributions to memory for emotion-laden stimuli as a function of valence.

In the present study, we examined regional gray matter correlates of immediate free recall and recognition memory of intentionally encoded positive, negative, and emotionally neutral words, respectively, in a larger group of middle-aged and older healthy adults. We conducted both region-of-interest (ROI) analyses for amygdalar and hippocampal volumes as well as whole-brain voxel-based morphometry (VBM) for examining possible associations between regional gray matter and emotional memory performance. Based on previous findings [54–55], we expected regional gray matter volume in the OFC and the ventromedial and ventrolateral PFC to be correlated with memory for negative words, when controlling for performance on positive and neutral words. As for behavioral results, based on previous findings [8], we did not expect to find a positivity bias in the memory tasks, because the present task required intentional memory encoding.

## Materials and methods

### Participants

The ethics committee of the Hospital District of Southwest Finland approved the study protocol. All participants gave written informed consent for participation in keeping with the Declaration of Helsinki and its later amendments. Altogether 49 monolingual native Finnish-speaking community dwellers aged 50 to 79 years with normal hearing, normal-to-corrected vision (eye glasses were permitted), and normal color vision took part in this study. They were recruited via an advertisement in a Finnish regional newspaper. To check for fulfilment of the inclusion criteria, a telephone interview was conducted prior to taking part in the study. Exclusion criteria included earlier or current neurological illness, a history of traumatic brain injury involving concussion, loss of consciousness, and/or post-traumatic cognitive dysfunction, current psychiatric diagnosis, current use of psychotropic medication, a history of psychoactive substance abuse, and having a close relative suffering from schizophrenia.

A further inclusion criterion was normal cognitive functioning, defined as a Clinical Dementia Rating memory box score of 0 (CDR) [59], a Mini-Mental State Examination score of at least 25/30 (MMSE) [60], and performance equal to or less than one standard deviation below the age-appropriate norms within a cognitive domain on a battery of standardized neuropsychological tests. The neuropsychological tests included Wechsler Adult Intelligence Scale-III subtests Similarities, Block Design, Digit Span, and Digit Symbol [61], Object Memory Test (naming, immediate and delayed free recall) [62], Wechsler Memory Scale-Revised subtests Logical Memory I and II, and Verbal Paired Associates I and II [63], Boston Naming Test [64], Controlled Oral Word Association Test (letter fluency, category fluency) [65], Trail Making Test [66], Stroop Color and Word Test [67], copy of Rey-Osterrieth Complex Figure Test [68], Clock Drawing Test (from the Consortium to Establish a Registry for Alzheimer’s Disease, CERAD) [69], and drawing of three-dimensional figures [70]. A minor decline on an individual subtest was allowed as a sign of intra-individual variation, as long as performance within that cognitive domain as a whole fulfilled the criterion [71]. Three participants were excluded due to a failure to fulfil this criterion.

Data from 46 participants were included in this study. The final sample included 29 women (63.0%) and 17 men (37.0%) with a mean age of 62.54 years (*SD* = 8.15 years) and mean years of education of 13.55 years (*SD* = 2.79 years). All participants but one were self-reported right-handers, as determined by a cut-off score of at least 87 on a modified version of the Edinburgh Handedness Inventory [72]. For none of the participants did next-of-kin report cognitive impairment in everyday life.

None of the participants received monetary compensation, but they were provided with written clinical feedback based on their individual neuropsychological performance by an experienced clinical psychologist (Carina Saarela) and on the magnetic resonance imaging (MRI) scan by a neuroradiologist (Riitta Parkkola).

### Memory tasks and procedure

All participants underwent a neuropsychological assessment, an electroencephalogram (EEG) experiment, and a MRI scan. The behavioral data (memory performance) for the analyses in the present study were gathered by an immediate free recall task and a recognition memory task employed in the EEG experiment that was conducted within a week of neuropsychological testing. EEG was recorded during the administration of both tasks. The EEG results will be reported elsewhere.

The participant was seated in a comfortable armchair about 1.2 m from a TV screen. In the *immediate free recall* task the participant was instructed to silently read and memorize a total of 150 Finnish nouns that were presented in fifteen 10-word lists varying in emotional valence, i.e., five word lists of each word valence group, and to freely verbally recall the previous word list while a question mark was displayed on the screen. The 150 Finnish nouns were chosen from a pool of 420 nouns [73]. The word valence groups were created as follows: negatively valenced words (mean valence < 3.00 on a Likert scale ranging from 1 to 7); emotionally neutral words (mean valence = 3.60–4.30); positively valenced words (mean valence > 5.00). The word valence categories differed significantly with respect to their mean valence ratings (positive > neutral > negative, all *p*-values < 0.001). To be able to control for possible effects due to the emotional arousal elicited by these words, we attempted to match the valence categories in the encoding task for this variable using the estimates in Söderholm et al. (2013) [73]. However, the positive and neutral words were matched for arousal, *t*(71) = 1.19, *p* = .237, but the negative words were on average significantly more arousing, *M* = 4.48, *SD* = 0.64, compared to the positive, *M* = 3.81, *SD* = 0.73, *t*(96) = 4.94, *p* < 0.001, and the neutral, *M* = 3.67, *SD* = 0.36, *t*(77) = 7.84, *p* < 0.001, words. As this arousal-related bias originated from the original pool of 420 words [73], it could not be amended. All words were nouns in nominative singular, which is the morphologically simple dictionary form in Finnish. The valence categories were matched for word length in letters, surface frequency, lemma frequency, bigram frequency, initial trigram frequency, and final trigram frequency. The mean values per item can be found in the supplementary material to Söderholm et al. (2013) [73]. They were originally taken from an unpublished extensive database of written Finnish (the Finnish newspaper Turun Sanomat published between 1^st^ March 1994 and 30^th^ June 1996, including 22.7 million words) using the computerized WordMill Lexical Search Program [74]. The selected 150 nouns had a surface frequency value of 0.04–83.96 per million, indicating low to medium frequency range. The word length of the nouns had already in the 420-word pool been limited to a range of 5 to 9 letters, because word length has been shown to affect memory performance [75].

The *immediate free recall* task consisted of a short practice run to familiarize the participant with the experimental procedure and a study run. The study run included 15 trials with ten words each. The words were presented only once. The presentation order of the words within each study run list and the presentation order of the lists were pseudorandomized using a 3 by 3 format to avoid any order effects. The only restriction during the randomization procedure was that there could be no more than two word lists of the same emotional valence presented in succession. The nine presentation orders were alternated on a participant-by-participant basis. The words were shown for 2000 ms followed by a 3000 ms interval. After each study run list, a prompt for the immediate free recall of the previous word list appeared on the screen for 60 s. The researcher documented the order in which the participant recalled the words and possible errors.

After the immediate free recall task, two tasks were administered to prevent the participant from rehearsing the stimuli. First, the participant was asked to count backwards aloud starting from 150. Counting was interrupted after 30 s. Second, a five-minute 0-back task with consonants was administered. The function of the 0-back task was also to familiarize the participant with the response pad. The time lag between the immediate free recall and recognition memory tasks was approximately ten minutes.

After the 0-back task, memory for the words in the immediate free recall task was investigated using an old-new *recognition* task that included making yes-no confidence judgments. The task was to identify the 150 target words from the immediate free recall task from among 300 randomly presented words, half of which were the target stimuli from the immediate free recall task, half new distractor stimuli. The 150 distractors were chosen from the same 420-word pool as the target stimuli [73]. The targets and distractors were matched on all emotional and psycholinguistic variables: valence, arousal, word length, surface frequency, lemma frequency, bigram frequency, initial trigram frequency, and final trigram frequency. Moreover, the distractors were chosen based on semantic relatedness, in that words that were closely semantically related to the target words were preferred. Matching of the distractor words on emotional and psycholinguistic features for the distractor valence categories was equally successful as for the targets, apart from the arousal variable due to the reasons stated above. The three valence categories differed significantly with respect to their mean valence ratings (positive > neutral > negative, all *p*-values < 0.001). However, none of the distractor valence categories were matched for arousal: the neutral words were on average more arousing, *M* = 3.74, *SD* = 0.48, than the positive words, *M* = 3.42, *SD* = 0.57, *t*(98) = 3.01, *p* = 0.003, whereas the negative words were again significantly more arousing, *M* = 4.69, *SD* = 0.53, than both the positive, *t*(98) = 11.50, *p* < 0.001, and the neutral words, *t*(98) = 9.46, *p* < 0.001.

In each *recognition memory* trial, a word was displayed on the screen and the participant was instructed to make an old-new discrimination using a response pad. After each old-new discrimination, the prompt for the yes-no confidence judgment appeared on the screen. The participant was instructed to respond as swiftly and accurately as possible. The recognition task also consisted of a practice run to familiarize the participants with the experimental procedure and a study run. The study run included 300 trials. A word was shown up to 2000 ms, and then a black screen was displayed for 2100 ms, followed by the confidence judgment for a maximum time of 1500 ms.

### MRI image acquisition

MRI scanning was conducted within 21 weeks of the EEG experiment (mean interval = 13.4 weeks, *SD* = 5.8 weeks). MRI was performed with a 3T scanner (Verio, Siemens Medical Imaging, Erlangen, Germany) at the Department of Radiology, Turku University Hospital. The parallel acquisition technique (GRAPPA) was used in all sequences. A routine 12-channel head coil was used. T2-weighted images had TR (Repetition Time) of 5210 ms, TE (Echo Time) of 96 ms, FOV (Field-Of-View) 220 mm x 165 mm, 4 mm slice thickness, and a 30% gap between images. FLAIR sequence had TR of 5000 ms, TI (Inversion Time) of 1800 ms, TE of 395 ms, FOV 250 mm x 250 mm, voxel size 1 mm x 1 mm x 1mm, and 160 slices in total with go gap between slices. 3DT1 sequence had TR of 2300 ms, TI of 900 ms, TE of 3 ms, FOV of 256 mm x 240 mm, and FA (Flip Angle) of 9 degrees.

### MRI analyses

#### ROI analyses

The left and right amygdalae and hippocampi were *a priori* selected ROIs. The volumetric segmentation was performed with the Freesurfer image analysis suite (http://surfer.nmr.mgh.harvard.edu). Briefly, this processing included motion correction and averaging [76], removal of non-brain tissue using a hybrid watershed/surface deformation procedure [77], automated Talairach transformation, and segmentation of the subcortical white matter and deep gray matter volumetric structures [78–79]. The automatic labelling technique limits the analysis to regions specific to the hippocampus, excluding cortical areas, and identifies the amygdalae using the hippocampi as anatomical landmarks [78].

#### Voxel-based morphometry (VBM)

VBM analysis was conducted using the VBM8 toolbox (Christian Gaser, University of Jena, Jena, Germany; http://dbm.neuro.uni-jena.de/vbm/) implemented in Statistical Parametric Mapping software (SPM8, Wellcome Department of Cognitive Neurology, London, UK) running in Matlab 2011a (Mathworks Inc., Natick, MA) [80–83]. Briefly, the processing included high-dimensional DARTEL normalization to Montreal National Institute (MNI) space, image intensity non-uniformity correction, and segmentation to gray matter, white matter, cerebrospinal fluid, and three non-brain partitions. The gray matter images were modulated using Jacobian determinants derived from the normalization procedure and smoothed using an 8 mm Full-width-at-half-maximum (FWHM) isotropic Gaussian kernel. Total gray matter, white matter, cerebrospinal fluid, and total intracranial volumes were calculated from the native space images.

#### Statistical analyses

Two repeated measures analyses of variance (ANOVA) with valence as the within-subject factor (three levels) were performed separately for immediate free recall and recognition memory performance (S1 Dataset). The analyses were conducted using proportional scores for correctly recalled words at immediate free recall (number of correctly recalled words divided by the maximum score of 50) and for correctly recognized targets (i.e., hits) at recognition (number of hits divided by the maximum score of 50). The recognition memory scores comprised pooled responses regardless of confidence judgment. Preliminary correlational analyses showed that age was significantly correlated with immediate free recall scores only. Thus, age was included as a covariate solely in the immediate free recall ANOVA. Further preliminary correlational analyses revealed no statistically significant correlations between any of the memory measures and the positive affect or negative affect scores on a Finnish unpublished adaptation (Saarela et al. unpublished manuscript) of the Positive and Negative Affect Schedule (PANAS) [84]. The equality of variances at different levels of the repeated factor was tested using Mauchly’s test of sphericity. *Post hoc*-analyses comparing different levels of the within-subjects factor were performed using paired samples *t*-tests. The *t*-tests were Bonferroni-corrected for the number of comparisons conducted (α_corrected_ = 0.05 / 3 (valence)] = 0.017). All statistical analyses of the behavioral data were performed with SPSS version 21 (SPSS Inc. IBM Company, 2012).

The associations between memory performance and amygdalar and hippocampal volumes, respectively, were tested using hierarchical linear regression analyses (S1 Dataset). In the first step, whole brain total gray matter, gender, and age were used as covariates to control for the variability in head size and overall cortical volume, and the potential confounding effects of gender and age, respectively. In line with Kumfor et al. (2013) [55] and Mistridis et al. (2014) [54], the second step introduced the proportional scores for opposite valence and neutral words (when valenced words acted as dependent variables) or for positive and negative words (when neutral words acted as the dependent variable) as covariates to control for “baseline” episodic memory performance. The third step introduced left and right amygdalar or hippocampal volumes as predictors for memory performance. The amygdalae and hippocampi were not included in the same regression analyses to avoid multicollinearity. Statistical analyses using the ROI data were performed with SPSS version 21 (SPSS Inc. IBM Company, 2012).

Age, gender, and total intracranial volume were included as nuisance variables to all the voxel-wise multiple regression analyses. In the analyses conducted separately for the valence categories, memory performance with the two other valence categories were used as additional covariates to account for “global” episodic memory performance, in a manner akin to that employed in the hierarchical regression analyses above and in line with Kumfor et al. (2013) [55] and Mistridis et al. (2014) [54]. An absolute voxel value threshold of 0.1 was used to restrict the analyses to the brain gray matter regions. Statistical significance was set at family-wise error (FWE) corrected P less than 0.05 at cluster level. Anatomical regions included to clusters were defined using the Automated Anatomical Labeling (AAL) toolbox (http://ww.gin.cnrs.fr/AAL) [85]. The peak coordinates are presented in MNI standard space. The results were visualized using Mango software (version 4.0.1; Lancaster, Martinez, http://rii.uthscsa.edu/mango/).

## Results

### Participant characteristics

MRI visual rating score data for the group are provided in Table 1. No focal white matter lesions were observed in 39.1% of the participants [86]. Focal lesions were found for 52.1% of the participants. 8.7% of the participants exhibited beginning confluence of lesions. General atrophy was found in one participant [87]. Also, one participant exhibited age-related left hippocampal atrophy and two exhibited age-related right hippocampal atrophy (score 1) [88]. Frontal atrophy with a score of 1 [89] was seen in 8.7% of the participants, whereas one participant had a score of 2 [89].

**Table 1:** MRI visual rating scores.

MRI measure	Description	Older adults (*n* = 46)
Age-related white matter changes^a^	*M (SD)*	0.70 (0.63)
	Score/number of cases	0/18, 1/24, 2/4
Hippocampal atrophy (left)^b^	*M (SD)*	0.02 (0.15)
	Score/number of cases	0/45, 1/1
Hippocampal atrophy (right)^b^	*M (SD)*	0.04 (0.21)
	Score/number of cases	0/44, 1/2
General atrophy^c^	*M (SD)*	0.04 (0.21)
	Score/number of cases	0/44, 1/2
Frontal atrophy^d^	*M (SD)*	0.13 (0.40)
	Score/number of cases	0/41, 1/4, 2/1

The age-related white matter changes and degrees of atrophy were visually evaluated by a single rater (R.P.) on scales ranging from 0 to 3 or 4.

^a^ White matter lesions. Score 0 = no white matter lesions; 1 = focal lesions; 2 = beginning confluence of lesions; 3 = diffuse involvement of the entire region, with or without involvement of U fibers. Basal ganglia lesions. Score 0 = no lesions; 1 = 1 focal lesion (≥5 mm); 2 = > 1 focal lesion; 3 = confluent lesions. Wahlund et al. Stroke.2001; 32: 1318–1322. [86]

^b^ Scheltens et al. J Neurol Neurosurg Psychiatry. 1992; 55: 967–972. [88]

^c^ Victoroff et al. Neurology. 1994; 44: 2267–2276. [87]

^d^ Jokinen et al. Parkinsonism and Related Disorders. 2009; 15; 88–93. [89]

### Effects of emotional content on memory performance

Means and standard deviations for the proportional scores for each valence category at immediate free recall and recognition are reported in Table 2. Two repeated measures ANOVAs were performed to compare the effect of valence on immediate free recall and recognition memory performance, respectively. At *immediate free recall*, Mauchly’s test indicated no violation of the assumption of sphericity, χ^2^(2) = 0.96, *p* = 0.400. Thus, uncorrected ANOVA results are reported for this analysis. There was no main effect of valence, *F*(2,88) = 0.32, *p* = 0.726, *η*^*2*^ = .007. The covariate age significantly predicted memory performance overall, *F*(1, 44) = 5.44, *p* = 0.024, *η*^*2*^ = .11, such that increasing age was associated with poorer free recall performance. At *recognition*, Mauchly’s test indicated a violation of the assumption of sphericity, χ^2^(2) = 0.85, *p* = 0.025. As estimated epsilon (ε) was greater than 0.75, the Huynh-Feldt correction was used. The main effect of valence was significant, *F*(1.795, 80.768) = 22.23, *p* < 0.001, *η*^*2*^ = .33. Follow-up paired samples *t*-tests revealed that recognition memory performance for positive words was significantly higher than for either negative, *t*(45) = 2.53, *p* = .015, or neutral words, *t*(45) = 5.60, *p* < 0.001 (Table 2). Recognition memory performance for negative words was also significantly higher than for neutral words, *t*(45) = 4.52, *p* < 0.001.

**Table 2:** Means and standard deviations for proportional scores separately by valence category at immediate free recall and recognition.

Valence	Immediate free recall	Recognition
Positive	0.51 (0.12)	0.75 (0.13)
Negative	0.49 (0.11)	0.72 (0.13)
Neutral	0.51 (0.12)	0.65 (0.15)

### Associations between emotional memory performance and amygdalar or hippocampal volumes

Hierarchical regression analyses were performed using the volumes of each ROI to predict immediate free recall and recognition performance separately for each valence category to determine whether amygdalar and hippocampal volumes were associated with memory performance. Taking into account whole brain total gray matter, age, gender, and memory performance in the two other valence categories, these analyses yielded a single significant positive association between right amygdalar volume and recognition memory performance for negative words, β = .317, *t*(45) = 2.51, *p* = 0.017. However, as the simple bivariate Pearson correlation was non-significant and of decidedly lower magnitude than the standardized beta-value, *r* = .03, *p* = 0.422, the presence of suppression was suspected. In this regression model, introducing step 1 with the control variables age, gender, and total brain volume explained 10.8% of the variance in recognition memory scores for negative words, *F*_*change*_ (3, 42) = 2.82, *p* = 0.051. In step 1, only the association with the control variable gender approached significance, β = .318, *t*(45) = 1.94, *p* = 0.060. Adding the control variables of recognition memory performance for positive and neutral words in step 2 increased the amount of variance explained to a sizable 71.9%, *F*_*change*_ (2, 40) = 46.77, *p* < 0.001. Both control variables had significant positive associations with recognition memory for positive words, as could be expected: recognition memory for negative words, β = .539, *t*(45) = 4.93, *p* < 0.001; recognition memory for neutral words, β = .378, *t*(45) = 3.51, *p* = 0.001. In the final step, adding the predictors left and right amygdalar volumes to the analysis explained merely additional 2.9% of the variance in recognition memory performance for negative words, *R*_*adj*_^*2*^ = .748, *F*_*change*_ (2, 38) = 3.23, *p* = 0.051. In the final step, the squared semi-partial correlation (*sr*^*2*^) showed that the amount of unique variance in memory performance explained by right amygdalar volume was quite modest, *sr*^*2*^ *=* .035, compared to that explained by, e.g., recognition memory for negative words, *sr*^*2*^ = .154. We attempted to identify which variables acted as suppressors using the method of rerunning the regression analyses leaving out the various predictors one at a time, as suggested by Tabachnick and Fidell (2007) [90]. As none of these reruns resulted in the size of the omitted predictor’s beta-value approaching that of the Pearson correlation coefficient more clearly than the beta-value of some other predictor, a specific suppressor or suppressors could not be pinpointed. According to Tabachnick and Fidell (2007) [90], the inability to identify the suppressors is quite common in multiple regression analyses. These results indicate that there were no statistically significant associations between left and right amygdalar volumes and immediate free recall performance in any valence category or recognition memory performance for positive and neutral words. Furthermore, hippocampal volumes did not predict memory performance on any measure.

### Whole brain VBM results

**Fig 1:**
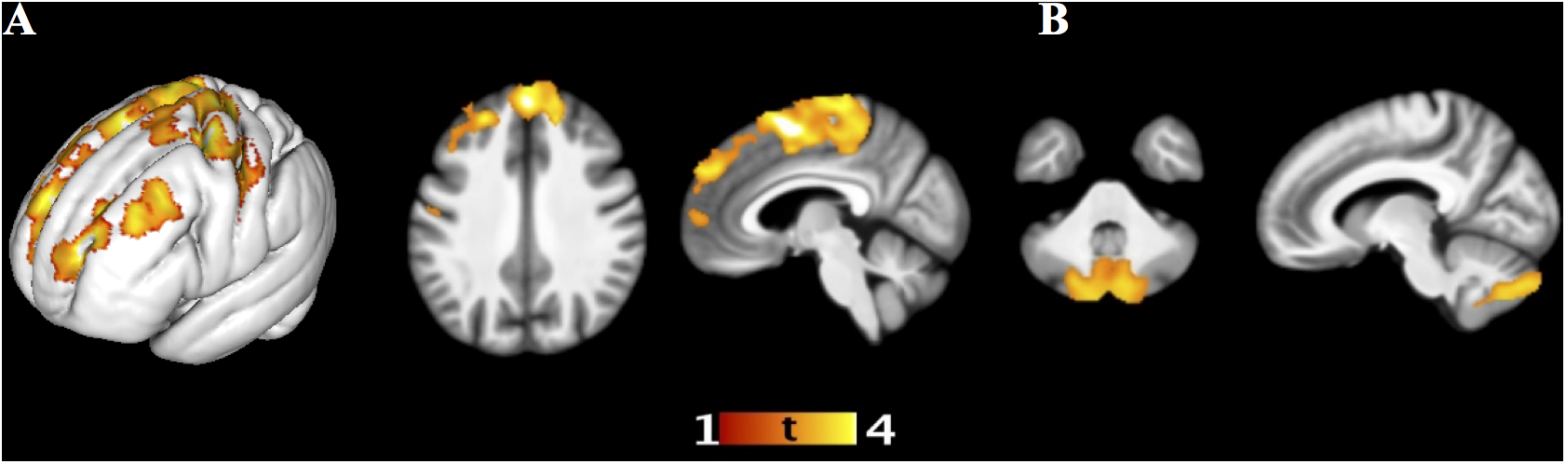
Immediate free recall and local gray matter volume. Association between local gray matter volume and (a) immediate free recall of negative words (negative association, height threshold T = 2.43, peak at 6 3 57 mm, cluster size 15015 voxels, P_FWE_ < 0.001, and peak at -27 50 25, cluster size 2681 voxels, P_FWE_ = 0.037), and (b) immediate free recall of positive words (positive association, height threshold T = 2.43, peak at 4–75–47 mm, cluster size 3627 voxels, P_FWE_ = 0.008). The statistically significant clusters are overlaid on the average normalized T1-weighted image of the studied sample.

The anatomical labeling of the clusters is presented in Table 3. Immediate free recall of negative words, taking into account memory performance for positive and neutral words, was negatively associated with regional gray matter volume in the frontal lobe, encompassing a large cluster in the dorsomedial PFC comprising the premotor and primary motor cortices in the superior frontal and precentral gyri, and a cluster in the left dorsolateral PFC (Fig 1A; Table 3). Higher immediate free recall performance for positive words, accounting for memory performance for negative and neutral words, was correlated with larger local gray matter volume in the cerebellum, specifically in a cluster centered mainly in bilateral Crus II in the mediolateral hemispheres of the posterior lobe (Fig 1B; Table 3). There was no statistically significant association between immediate free recall of neutral words and local gray matter volume.

**Table 3:** Anatomical region, %cluster, cluster size in voxels, peak coordinates (MNI), and significance level of all significant associations between local gray matter volume and memory for emotion-laden words.

Anatomical region	Laterality	%Cluster^a^	*k*^b^	Peak (*x*, *y*, *z*)^c^			Sig.^d^
				*x*	*y*	*z*	
*Immediate recall*: *negative words*							
Supplementary motor area	Right	20.4	15015	6	3	57	< 0.001
Superior frontal gyrus	Right	13.7					
Supplementary motor area	Left	9.3					
Paracentral lobule	Right	9.0					
Medial frontal gyrus	Right	8.1					
Precentral gyrus	Left	7.1					
Medial frontal gyrus	Left	5.8					
Paracentral lobule	Left	4.9					
Middle frontal gyrus	Right	4.6					
Middle frontal gyrus	Left	66.4	2681	-27	50	25	0.037
Superior frontal gyrus	Left	28.5					
*Immediate recall*: *positive words*							
Cerebellum Crus II	Right	27.7	3627	4	-75	-47	0.008
Cerebellum Crus II	Left	24.7					
Cerebellum lobule VIII	Right	6.1					
Cerebellum lobule IX	Left	5.8					
Cerebellum lobule IX	Right	4.4					
*Recognition*: *positive words*							
Calcarine sulcus	Right	25.0	8109	21	-49	-5	< 0.001
Lingual gyrus	Right	22.3					
Cuneus	Right	11.9					
Superior occipital gyrus	Right	9.6					
Cuneus	Left	8.6					
Calcarine sulcus	Left	5.2					
Fusiform gyrus	Right	4.4					

^a^ Percentage of total cluster size.

^b^ Cluster size in voxels.

^c^ Peak coordinates in MNI space.

^d^ Family-wise error (FWE) corrected P.

**Fig 2:**
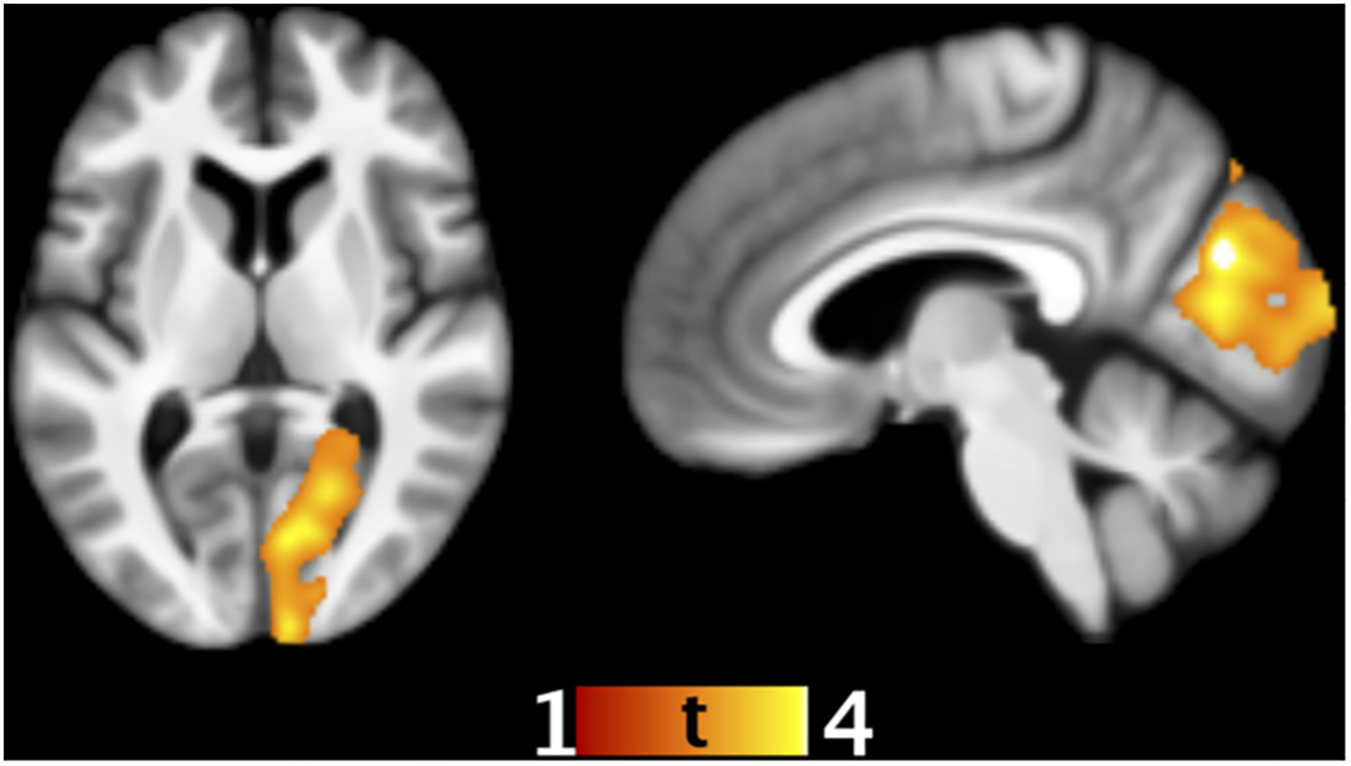
Association between recognition memory of positive words and local gray matter volume. The statistically significant cluster (negative association, height threshold T = 2.43, peak at 21–49–5 mm, cluster size 8109 voxels, P_FWE_ < 0.001) is overlaid on the average normalized T1-weighted image of the studied sample.

No statistically significant associations between recognition memory of either negative or neutral words and regional gray matter volume were observed. However, a significant negative association between recognition memory of positive words and local gray matter volume was found in the occipital lobe mainly in the cuneus, extending into the lingula and primary visual cortex (Fig 2; Table 3).

## Discussion

The present study sought to examine the associations between regional gray matter volume and memory performance for emotion-laden and emotionally neutral words on immediate free recall and recognition memory tasks in a sample of cognitively intact 50-79-year-old adults. Behaviorally, we found no effect of emotional content on immediate free recall performance, but the participants exhibited both the EEM and a positivity bias in recognition memory. In other words, recognition memory of both positive and negative words surpassed that of neutral words, and positive words were better recognized than negative words. In the hierarchical multiple regression analyses using ROI volumes, there were no statistically significant associations between hippocampal or amygdalar volumes and memory performance in any valence category after accounting for age, gender, whole brain total gray matter, and memory performance in the other valence categories. In contrast, the whole-brain VBM analyses, accounting for age, gender, total intracranial volume, and memory performance in the other valence categories, produced some novel statistically significant associations. Firstly, higher immediate free recall of negative words was associated with smaller regional gray matter volume in the frontal cortex, encompassing clusters in the dorsomedial and left dorsolateral PFC. Secondly, better immediate free recall of positive words was correlated with larger local gray matter volume in the mediolateral hemispheres of the posterior lobe of the cerebellum. Thirdly, higher recognition memory of positive words was associated with smaller regional gray matter volume in the occipital cortex, encompassing a large area in the cuneus, extending into the lingula, and thus comprising the primary visual cortex. No statistically significant associations were found for memory for emotionally neutral words or recognition memory of negative words.

### Effects of emotional content on immediate free recall and recognition memory

Contrary to expectations, the EEM was demonstrated only for recognition memory, but not for immediate free recall. This pattern for the EEM was somewhat surprising in light of a meta-analysis showing a medium EEM for free recall compared to a small EEM for recognition memory in older age [4]. Methodological differences may explain this discrepancy. For instance, the meta-analysis included both immediate and delayed conditions in free recall studies, such that delayed recall was included whenever immediate recall measures were unavailable.

In contrast to the expectation that no positivity bias would emerge here due to the fact that an intentional encoding paradigm was used [8], a positivity bias was seen in recognition memory. In a meta-analysis, a positivity bias in older age surfaced in studies using unconstrained processing instructions, such as free viewing in an incidental encoding paradigm, but not in studies using constrained processing instructions, such as intentional encoding [8]. However, the effect size for that finding was small. There are previous studies using intentional encoding instructions that have demonstrated preferential memory performance for positive words in healthy older adults [11–12, 91]. Furthermore, Kensinger (2008) reported a positivity bias for both incidentally and intentionally encoded words, but only for low-arousing words [12]. Taken together, these findings indicate that the mechanisms driving the positivity effect in memory in middle-aged and older adults are complex and not yet fully understood.

### No association between amygdalar or hippocampal volume and memory for emotion-laden words

In line with previous work with middle-aged and older healthy adults [49–50, 53], no associations between amygdalar volume and memory performance were found in the present study, save from the positive association with recognition memory of negative words that stemmed from statistical suppression. We could not identify which covariates acted as suppressors, which is quite common when suppression is encountered in multiple regression analyses [90]. Still, as the association emerged as a result of suppression, it will not be discussed further. Most earlier studies on amygdalar volumetric correlates of memory for emotion-laden stimuli reporting positive findings have used mixed samples of dementia patients and healthy controls [54–56], leading to greater variance in measures, and thus increasing the statistical power to detect correlations [92]. Indeed, the scatterplots representing the statistically significant relationships between memory performance and amygdalar volume in the supplementary material in Mistridis et al. (2014) suggest restricted variance for both measures when looking at the separate groups [54]. Furthermore, there are mixed findings regarding the existence of age-related decline in amygdalar volume [45–46, 57–58, 93], which may inadvertently affect variability in measures in different studies.

Studies on the association between hippocampal volume and memory for emotion-laden stimuli in middle-aged and older adults have produced mixed results. In line with Landré et al. (2013) [50] and Schultz et al. (2009) [53], but in contrast to Guzmán-Vélez et al. (2015) [49], hippocampal volume did not predict emotional memory in the present study. The inconsistency of the hippocampal volumetric associations may be explained by methodological differences between studies, such as group composition, stimulus type, memory measures, and methods of analysis. A positive relationship might have been expected, as a meta-analysis on the relationship between hippocampal volume and memory performance in healthy individuals over the lifespan did demonstrate a weak positive relationship in older age [47]. However, the main finding for the older age groups was increased variability in both hippocampal volume and memory performance. Also, the choice of statistical methods pertaining to adjusting for age and brain size was shown to impact on the results particularly in the older group [47]. Null findings on the relationship between hippocampal volume and memory performance are not uncommon in normal aging [94]. These null findings have been explained in terms of little or no functional effects of the small volumetric changes in the hippocampi accompanying healthy aging, as the changes may have non-pathological developmental origins [95]. By comparison, the positive relationships seen in neuropathology have been taken to imply that larger hippocampal volumes equate to larger remaining segments of functional neural tissue, leading to better performance, also called the ‘bigger is better’ hypothesis [95].

### Immediate free recall of negative words is associated with local frontal gray matter volume

Contrary to previous research [54–55], our findings pertaining to frontal lobe volumetric associations for memory for negative words were not localized to the OFC and ventromedial and ventrolateral PFC. Instead, immediate free recall of negative words was negatively correlated with regional gray matter volume in the dorsomedial PFC and the left dorsolateral PFC. A possible explanation for the different localization may be that previous studies have been conducted with mixed samples of patients with neurodegenerative conditions and healthy older adults, and the present study has focused on healthy middle-aged and older adults. We propose that the negative correlation could be construed as reflecting of the involvement of these frontal areas in the cognitive control of emotion [32–33, 40–41] and in self-referential processing [34, 38–39].

There are two main theoretical accounts for explaining the observed age-related differences in the preferences for emotional stimulus content. One is the socioemotional selectivity theory [96], which proposes that the preferences are driven by chronically activated motivational goals via top-down control processes [8]. In middle and older adulthood, when the time perspective is more constrained, goals associated with emotional meaning and current well-being gain importance, whereas goals associated with preparation for the future lose importance [6–7, 96]. In middle-aged and older adults, the preference for processing positive information or the reduced preference for processing negative information is thought to stem from a natural inclination to use controlled processing strategies to promote emotion regulation with the aim of achieving the goals associated with current emotional well-being [6–8, 96]. The other theoretical account explains the positivity effect in terms of age-related cognitive or neural deficits [7, 97–98]. Labouvie-Vief et al. (2010) suggested that positive information is less cognitively demanding than negative information and that age-related cognitive decline therefore may cause the positivity bias in older age [97]. Cacioppo et al. (2011) put forth the aging-brain model, which postulates that older adults exhibit decreasing amygdalar activation specifically to negative stimuli [98]. This would entail the attenuation of emotional arousal to negative stimuli, which, by extension, would lead to worse memory for negative stimuli in older age [98]. The socioemotional selectivity theory suggests that the positivity effect is driven by top-down control processes, whereas the deficit-based theories propose that it is driven by bottom-up automatic processes [7]. There is still no consensus on which of these theories should be considered dominant in explaining the positivity effect. However, an inherent confound in functional neuroimaging research on age-specific amygdalar activation during emotional processing to be noted in this context is the relatively slow development of the hemodynamic response [99]. It has therefore been proposed that the amygdalar activation that has been measured in functional magnetic resonance imaging may reflect the result of top-down modulation by prefrontal mechanisms rather than bottom-up arousal-driven modulation via the visual cortex [99]. A recent study seemed to offer better support for the socioemotional selectivity theory than for the deficit-based account, but neither theory received full support [100]. However, the present results cannot be used as proof for either account. As the present structure-function correlation was negative and localized to the frontal lobes, we opted for the socioemotional selectivity theory and considered an explanation involving cognitive control processes as being more feasible than the aging-brain model with its emphasis on degraded amygdalar functioning and automatic processing.

The PFC areas involved in cognitive control functions influence what information one attends to, and how the meaning of a stimulus is interpreted, thereby regulating the activity so that it is congruent with the implicitly activated goal [41]. However, the cognitive control hypothesis may be considered argumentative, because the PFC areas that tend to engage in emotion regulation [32–33, 40–41] partly overlap with the PFC areas that decline with age [57]. Also, cognitive control tends to become less efficient with advancing age [6–7]. However, the positivity effect in memory has been shown to emerge only for older adults with high levels of cognitive control, whereas those with low levels of cognitive control exhibited a negativity bias akin to that of the young adults [6]. Also, age-related shifts have been demonstrated in the preferences for regulatory strategies towards less cognitively demanding ones that are subserved by more preserved brain regions [57]. This may be construed as evidence for the cognitive control hypothesis. As for arousal-related processing, EEM for nonarousing information is proposed to be based on controlled processing, and EEM for arousing information on automatic processing [12]. The arousal-driven automatic processing would function as automatic capture of attention [3], hypothesized to occur because of the evolutionary benefits of the facilitated or prioritized processing of arousing stimuli [2]. Controlled processing would entail semantic elaboration or self-referential processing of the nonarousing information, which middle-aged and older healthy individuals would use to promote a more positive emotional state. We suggest that, provided that there is a positive correlation between the structural integrity and functional efficiency of these frontal areas, this negative association between regional frontal gray matter volume and immediate free recall of negative, relatively high-arousing words could indicate that reduced regional gray matter volume in these areas may have served to attenuate the cognitive control necessary to represent and actively maintain the implicitly activated goals. Consequently, the regulatory processing needed to achieve goal-congruent behavior was disabled and arousal-driven automatic processing was enabled, ultimately resulting in enhanced memory for these stimuli by virtue of their attention-grabbing effect.

Support for this hypothesis can also be found in functional brain imaging studies indicating that older adults seem to engage more neurocognitive resources to the processing of positive information and to down-regulate emotional responses to negative information, particularly in frontal areas [101–103]. As for the lack of a correlation with the ventromedial PFC, Bechara et al. (2000) showed that patients with ventromedial PFC lesions but not basal forebrain lesions exhibited normal EEM despite abnormal reactivity to emotional stimuli [104], suggesting that the EEM is not primarily subserved by this brain region. The negative association between memory performance and frontal gray matter volume is not unique to our study. Gautam et al. (2011) demonstrated that in older adults, smaller volume and cortical thickness of the lateral PFC was associated with better performance on a verbal memory composite score consisting of immediate and delayed free recall of a word list [95]. This suggests that this negative structure-function relationship may apply to verbal episodic memory in general.

### Immediate free recall of positive words is associated with local cerebellar gray matter volume

Larger local gray matter volume in a cerebellar cluster centered in bilateral Crus II of the mediolateral hemispheres of the posterior lobe was associated with better immediate free recall of positive words. At first glance, the cerebellar localization seems unexpected, as this structure has not been included in the neural network underpinning the EEM [2, 23, 29], or implicated in previous studies on regional gray matter correlates of memory for emotion-laden stimuli [54–55]. However, a closer look at some functional neuroimaging studies on memory for emotion-laden stimuli reveals that cerebellar activations during encoding and retrieval have been observed, but not discussed, most likely because the focus was on other brain structures [31, 105]. Still, during the past decades it has become increasingly evident that the cerebellum is an integral part of distributed neural networks subserving higher cognitive functions and emotional processes over and above the sensorimotor functions that have traditionally been attributed to it [106–113]. The cerebellum is thought to contribute to these neural networks with a modulatory function via a number of neural pathways connecting it with cortical and subcortical cerebral structures [107, 109]. The connectivity pattern converges with clinical and neuroimaging evidence on cerebellar functional topography [106, 109–111]. In broad terms, the anterior cerebellum and posterior lobule VIII are thought to be primarily involved in sensorimotor functions, the posterior vermis in emotional processing, and the posterior lobe, particularly lobules VI and VII [Crus I, Crus II], in higher cognitive functions, such as executive functions, language, episodic memory, working memory, and visuospatial processing [111–112].

Lesion studies [112, 114] and functional neuroimaging studies [115–117] have implicated the cerebellum in a variety of learning and memory tasks, also including fear conditioning [118] and recognition memory of emotion-laden stimuli in young adults [31, 119]. To the best of our knowledge, neither functional nor structural neuroimaging studies demonstrating cerebellar contributions to memory for emotion-laden words in middle-aged and older adults have been reported. This study is the first one to report an association between posterior cerebellar gray matter volume and immediate free recall of positive, relatively low-arousing words. As previously stated, the preferentially enhanced memory for positive, low-arousing stimuli that is encountered in middle-aged and older healthy adults has been hypothesized to be driven by chronically activated motivational goals to promote emotional well-being via cognitive control processes [8], such as semantic elaboration or self-referential processing of nonarousing information. Provided that larger cerebellar volume indicates stronger functional efficiency, it may be that larger regional gray matter volume in the mediolateral hemispheres of the posterior cerebellum is related to better memory for positive, low-arousing stimuli through the conjoint effect of the involvement of these areas in cognitive control [107–108, 113, 120–121], inhibitory control on arousal [122], facilitation of reward system functioning [123–124], and self-relevant and self-referential processing [38–39] in middle and older adulthood. Also, lobule IX seems to be part of a functional resting state network, the default mode network, which has been implicated in episodic memory and self-reflection [113].

### Recognition memory of positive words is associated with regional occipital gray matter volume

Smaller regional gray matter volume in the cuneus and lingula of the occipital lobe, specifically in an area corresponding to the primary visual or striate cortex (BA 17, V1), was correlated with better recognition memory of positive words. Both the localization and direction of the association were quite surprising. Meta-analyses on functional neuroimaging studies have found preferential activation to emotion-laden stimuli in the occipital areas V2 to higher visual association cortices (BA 18 and beyond), not in V1 (BA 17) [27, 125]. In a study on the relationship between EEM in story recall of narrated slides and gray matter intensity, EEM was correlated with gray matter intensity in BA 18 [56]. In studies on the incidental encoding of emotion-laden stimuli, the primary visual cortex has usually activated in response to any stimuli regardless of their emotional content [21–22]. However, as some studies have reported enhanced occipital activation to memory for emotion-laden stimuli without disclosing the exact localization of the cluster [20, 31], it is unclear whether the primary visual cortical activation is unspecific to emotion-laden stimuli or not. In fact, enhanced activation of the cuneus (BA 17) has been observed during the emotional discrimination of faces in young adults [102]. Consequently, it is difficult to account for this finding. We welcome further studies including replications to gain a more thorough understanding of the associations between recognition memory of emotion-laden words and regional gray matter volume in middle-aged and older adults.

### On the interpretation of structure-function relationships in a cognitively intact middle-aged and older sample

The interpretation of our results rests on theories on the mechanisms that drive memory for emotion-laden stimuli in middle-aged and older adults. This mode of interpretation could be construed as problematic, as age was controlled for in the VBM analyses. Therefore, the results could be regarded as age-invariant, which would preclude age-specific interpretations. However, the fact remains that the sample represented middle-aged and older adults. When conducting studies on gray matter volumetric correlates of behavior, the results are known to be dependent on the age of the participants and the presence of brain pathology [48]. This is thought to reflect that the microstructural mechanisms underlying local gray matter volume as measured by VBM are likely to be different in young adults, normally aged adults, and people with neuropathological conditions [44, 48]. After all, it is not precisely known what aspects of microstructure and which cellular events contribute to local gray matter volume as measured by VBM [44]. Also, the effect of using age as a covariate was assessed in a study, where a negative correlation between PFC regional volume and cognitive measures in a sample of healthy older adults was found to hold up after controlling for age [126].

The directionality of some of our results is surprising, as the general assumption is that the size of brain structures and their functional efficiency (in terms of both functional activation and cognitive/behavioral efficiency) are positively correlated. However, the relationship between regional gray matter volume and functional efficiency seems to be quite complex. It has been shown that this relationship varied according to memory process (encoding, retrieval) and brain region, even within the PFC, in older individuals [127]. For example, local gray matter atrophy partly accounted for reduced occipital activation at encoding, and for left prefrontal, parietal and right cerebellar enhanced activation at retrieval in older adults as compared to young adults [127]. Stern et al. (2005) stated that the processing efficiency of cortical structures may be related not only to their size, but also to their functional efficiency, implying that when less tissue is related to stronger activation to produce higher levels of a certain behavior, a compensatory mechanism may be at play [128]. To approach a resolution to these conundrums would entail amending some of the limitations to our study, such as including young adults and patient groups as well as studying both structure-function relationships and the activation of the implicated brain areas during task performance.

### Limitations

This study has limitations affecting the generalizability of the results. First, the study was conducted using a convenience sample of older middle-aged and older adults, and no young adults were included. However, the group was quite representative of its age segment in terms of gender and educational attainment [129]. Also, the sample size was modest, but still exceeding sample sizes in most previous studies. The time lapse between the behavioral tasks and the MRI scan was quite long, 13.4 weeks on average.

Another limitation is the use of standardized valence and arousal evaluations to create word valence categories, as it has been shown that effects of emotional stimulus content on memory performance may vary depending on whether objective or subjective evaluations are used [130]. This could be of particular importance to the outcome of the behavioral analyses. Related to this, a further potential pitfall of our study may be our inability to disentangle the effects of valence and arousal on memory performance and in the explanations of our structure-function correlational findings, as we could not match the negative and positive words for arousal. However, it is well known that the dimensions of valence and arousal tend to be inter-correlated, especially regarding negative stimuli [73, 131].

Moreover, the use of automated software-based tracing of the amygdala may be considered a limitation, as previous studies have shown that the identification of the amygdala using even very sophisticated software compared to manual tracing presents with a challenge [49, 132]. However, the algorithm for the detection of amygdalar volume of the automatic labelling technique used in this study is considered as quite reliable and valid [78].

### Conclusions and future directions

In conclusion, our study demonstrated the presence of both EEM and a positivity bias in recognition memory, but not immediate free recall, of intentionally encoded words in a sample of cognitively intact 50-79-year-old adults. Structure-function correlational analyses revealed no statistically significant associations between amygdalar or hippocampal volume and the memory measures. Whole-brain VBM analyses yielded associations between memory for emotion-laden words and regional gray matter volume in the dorsomedial and dorsolateral parts of the frontal cortex, and in the cerebellum, suggesting that memory for emotion-laden words in healthy middle and older adulthood is dependent on the structural integrity of brain areas directly subserving cognitive control processes. Also, a surprising association between primary visual cortex volume and recognition memory of positive words was revealed. The results suggest that cognitively intact middle-aged and older adults show distinctive features in the structure-function relationships for memory for emotion-laden stimuli. As a whole, much remains to be learned in this field of research, especially about the effects of normal, neurologically healthy aging on the structural brain correlates of emotional memory processes.

The explanation to our results in terms of the neural substrates of the differential effects of automatic and controlled processing on memory for emotion-laden words clearly warrants future studies that should include young adults as well as patients with neurodegenerative disorders. Furthermore, the intricate relationship between neural structure and function would be best examined in an event-related experimental paradigm, which would enable assessment of the impact of structural correlates on the functional efficiency of the implicated brain areas.

Hogan et al. (2011) [133] and Paul et al. (2009) [134] showed that positive correlations between cerebellar volume and various cognitive measures in older age disappeared when frontal lobe volume was accounted for, indicating a primary role for age-related changes in the frontal lobe in driving age-related cognitive changes, and by extension a primary role for the frontal lobe in subserving these cognitive functions. This indicates that it would be fruitful to extend this approach to our research area with a larger sample that would permit more statistical power to detect such relationships. Finally, as the cognitive control explanation to our findings rests upon the existence of anatomical and functional connections between these various brain structures, future studies using diffusion tensor imaging and functional connectivity approaches would also be warranted.

## Supporting information

S1 Dataset Demographic, behavioral and ROI volumetric data. (SAV)
